# Modelling Asphalt Overlay As-Built Roughness Based on Profile Transformation—Case for Paver Using Automatic Levelling System

**DOI:** 10.3390/s24072131

**Published:** 2024-03-27

**Authors:** Rodrigo Díaz-Torrealba, José Ramón Marcobal, Juan Gallego

**Affiliations:** Departamento de Ingeniería del Transporte, Territorio y Urbanismo, Universidad Politécnica de Madrid, 28040 Madrid, Spain

**Keywords:** as-built roughness, pavement rehabilitation, asphalt overlay, road profile, paver screed, automatic levelling, international roughness index

## Abstract

The as-built roughness, or smoothness obtained during pavement construction, plays an important role in road engineering since it serves as an indicator for both the level of service provided to users and the overall standard of construction quality. Being able to predict as-built roughness is therefore important for supporting pavement design and management decision making. An as-built IRI prediction model for asphalt overlays based on profile transformation was proposed in a previous study. The model, used as basis for this work, was developed for the case of wheeled pavers without automatic screed levelling. This study presents further development of the base prediction model, including the use of an automatic screed control system through a long-distance averaging mobile reference. Formulation of linear systems that constitute the model are presented for the case of a wheeled paver using contactless acoustic sensors set-up over a floating levelling beam attached to the paver. To calibrate the model, longitudinal profile data from the Long-Term Pavement Performance SPS-5 experiment was used, obtaining a mean error of 0.17 m/km for the predicted IRI. The results obtained demonstrate the potential of the proposed approach as a modelling alternative.

## 1. Introduction

It has been demonstrated that surface roughness achieved in pavement construction, or as-built roughness, is of great importance for both future roughness progression and overall life-cycle pavement performance [1,2]. Reduced initial roughness in pavements will extend their service life, improve quality for users over time, and the highway agency will require less time and money for their maintenance. This will also reduce the inconveniences and risks that drivers encounter when passing through construction zones [3].

Determining the effectiveness of different treatments is a critical factor when selecting pavements maintenance and rehabilitation strategies; therefore, estimating as-built roughness is an important concern for designers, contractors, or any highway agency [4]. As-built roughness prediction can be used by contractors to evaluate the ride quality that will be achieved under a specific set of construction parameters, or by designers to assess the impact of their design choices on smoothness. Appropriate assessment of as-built roughness can influence highway agencies’ decision making about the necessity and type of maintenance required. Furthermore, as-built roughness is required as an input variable on many of the IRI performance prediction models commonly used in pavement-management systems [5,6].

According to the information mentioned above, predicting as-built roughness is essential for supporting decision making and optimization in pavement construction and management. A comprehensive as-built roughness prediction model may reduce the life-cycle costs of highways and improve the service quality to the travelling public. In addition, budget savings and environmental improvements can also result from the selection of cost-effective paving solutions.

### 1.1. Research Problem

Even though as-built roughness assessment is essential, there has not been much research carried out on it in the literature, and empirical methods have traditionally been used to investigate this problem. Díaz-Torrealba et al. [7] conducted a review and analysis of prediction models for as-built roughness in asphalt overlay rehabilitations. The findings indicated that existing IRI prior to overlay construction is the most used variable in the literature to explain as-built roughness IRI; based on this approach, several regression models have been proposed, although with insufficient capability to account for the observed variability in smoothness obtained during pavement construction.

In this context, when considering the roughness reduction effects of overlays, some studies have indicated that the main wavelengths that contribute to the existing IRI must also be taken into account [8,9,10]. However, because IRI does not provide specific information on roughness nature (wavelengths that make up profile defects), the available empirical models have not adequately taken these effects into account.

Based on their findings, Díaz-Torrealba et al. [11] proposed an as-built IRI prediction model based on profile transformation, in terms of wavelength content modification, as a result of the paver-levelling effect during laydown. The cited model was developed for the case of wheeled pavers without automatic screed levelling.

The asphalt paver working principle can be used to explain the previously described relationship between the wavelength content of the existing profile and the smoothness achieved during construction. When placing the asphalt mix, the levelling response of the paver screed is related to profile defects nature in a such a way that the longer the undulation on the surface being paved, more accurately the screed will replicate these undulations, i.e., the screed decreases its levelling efficiency as the wavelength of the defects causing the irregularity increases [12,13]. For this reason, pavers have incorporated the use of leveling beams (long reference averaging) and automatic control systems to regulate the screed tow-point position during paving and in this way compensate for the lowest leveling efficiency at longer wavelength defects.

This study presents further development of the cited prediction model [11] to include into de analysis the use of an automatic screed control system through a long-distance averaging mobile reference. The purpose of the developed model is to provide a tool to evaluate the efficiency of a certain overlay design on roughness correction, so project adjustments can be made before construction takes place, for example, to assess the cost benefits of placing the overlay in one or several lifts, help with the selection of paver type and size, examine the effects on roughness of changing the overlay thickness, or evaluate smoothness effect of using automatic levelling systems.

### 1.2. Study Approach

The model was developed using linear systems analysis techniques, utilizing the Laplace transform and the capabilities of Matlab^®^ software (version R2018b) to handle transfer functions. The following factors that affect the longitudinal profile transformation during the asphalt mix laydown were taken into consideration when formulating the equations describing an idealized automatic screed control system: (1) long-distance averaging mobile reference, (2) screed position error in relation to the reference and control signal, (3) solenoid valve oil-flow response to control signal, and (4) tow-point hydraulic cylinder position correction.

The model response was calibrated to minimize the prediction error between the real IRI values observed after works and IRI calculated from longitudinal profiles resulting from the paving simulations for the same sites.

Longitudinal profile surveys and construction information available for asphalt overlay projects in the SPS-5 experiment of the Long-Term Pavement Performance (LTPP) program were used as data sources for calibration. The model was developed for the case of wheeled pavers with the use of assisted levelling system.

## 2. Background

According to the asphalt layers construction process, the surface quality obtained during paving will mostly depend on the force balance acting on the paver screed [14,15]. The screed, which is attached to the tractor unit at the “tow point” by two lateral tow arms, is the mechanical tool of the paver used for placing, levelling and pre-compaction of the pavement material. The mat thickness and profile are established as the tractor tows the screed forward and the asphalt mix flows under it causing the screed to lift or “free float” over the mix. Because of this working principle, the screed can pivot around the tow points and smooth out the underlying surface irregularities as the mix is being placed. In this regard, the screed will add more asphalt mix to the depressions and less over the humps, compensating the bottom layer roughness and creating a new longitudinal profile.

### 2.1. Base Model for Paver Screed Response

**Figure 1:**
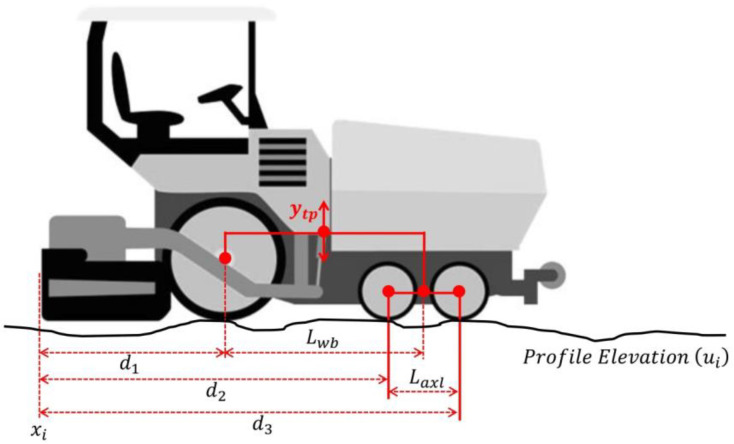
Calculation of tow-point trajectory [11], Reprinted by permission of the publisher (Taylor & Francis Ltd., http://www.tandfonline.com/).

The basis for this work is the as-built IRI prediction model proposed by [11]. The model simulates the paver levelling effect during laydown by means of profile transformation and was developed for the case of wheeled pavers without automatic screed levelling. Four sub-models are integrated into the main model to describe an idealized asphalt laydown construction process. A summary of the base model is presented as follows:

(1) Motion of wheeled pavers over the surface being paved and its effect on tow-point trajectory. The tow-point position during paving (y_tp_) was calculated as the average height between rear driving wheel and front bogie axles. Likewise, the front bogie axle position was calculated as the average height between the two oscillating wheels of the bogie (see Figure 1). Equation (1) presents the sub-model for tow-point trajectory as a result of paver motion.

(1) $$y_{tp}=\frac{1}{2}\left[u\left(i+d_{1}\right)+\frac{u\left(i+d_{2}\right)+u(i+d_{3})}{2}\right]$$

where

y_tp_: Tow-point elevation for each screed position x_i_ (m).

d_1_: Distance from screed position x_i_ to rear driving wheel axle (m).

d_2_: Distance from screed position x_i_ to oscillating bogie rear wheel axle (m).

d_3_: Distance from screed position x_i_ to oscillating bogie front wheel axle (m).

i: Each of the ‘n’ elevation points of the longitudinal profile ‘u’ (i = 1, 2, …, n).

In Equation (1) the profile elevation ‘u’ is referenced with respect to the back of the paver (screed trailing edge).

(2) Kinematic model for screed trajectory. This model represents the screed response time to changes in the tow-point position, which is commonly accepted as five tow lengths. Considering that all the forces acting on the screed stay constant, the screed trajectory in response to tow-point changes was reduced to calculation of its kinematic movement. The transfer function H_1_(s) for this system is given by the relationship between the Laplace transforms of the response y_1_(s) and input signal to the system, which is the tow-point trajectory y_tp_(s). Equation (2) presents the sub-model for the vertical position of the screed in response to the tow-point trajectory.

(2) $$\frac{y_{1}(s)}{y_{tp}(s)}=H_{1}\left(s\right)=\frac{1}{1+\tau s}$$

where

τ: Time constant (L_b_/V) (s).

L_b_: Tow arm length (m).

V: Paver speed (m/s).

(3) Dynamic model for screed vibration. The vertical movement of the tow point and its effect over the screed will not be the only factor in determining the surface finish. These factors were incorporated into the model as a local screed vibration caused by short-wave roughness on the longitudinal profile under the screed plate. This dynamic response will occur independently of the screed vertical position and its result will overlap the trajectory.

For the system response to be independent of the road vertical alignment, the input signal used was the first difference u_f_(t) of the existing profile u(t), or detrended profile, calculated over intervals equals to the screed plate width L_t_ (m). This filtering was implemented by the transfer function in the Laplace domain presented in Equation (3).

(3) $$u_{f}\left(i\right)=u\left(i\right)-u\left(i-L_{t}\right)\Rightarrow \frac{u_{f}(s)}{u(s)}=1-e^{-\left(\frac{L_{t}}{V}\right)s}$$

In Equation (3), the profile elevation ‘u’ is referenced with respect to the screed-plate front edge. The transfer function of the system H_2_(s) is defined as the relationship between the Laplace transforms of the system response y_2_(s) and the input signal or excitation, which in this case corresponds to the surface profile detrended and smoothed over the width of the screed plate u_f_(s). Equation (4) presents the sub-model of screed vibration in response to short-wave roughness under the screed plate.

(4) $$\frac{y_{2}(s)}{u_{f}\left(s\right)}=H_{2}\left(s\right)=\frac{cs+k}{ms^{2}+cs+k}=\frac{2\zeta \omega_{n}s+\omega_{n}^{2}}{s^{2}+2\zeta \omega_{n}s+\omega_{n}^{2}}$$

According to the above, the vertical dynamics of the system depends on the screed mass (m), as well as on the stiffness (k) and damper (c) features of the model, that simulate the stiffness of the asphalt mix flowing under the screed and its damping effect. For calibration purposes, Equation (4) was expressed in its standard form, given by the undamped natural frequency of the system (ω_n_) and the damping ratio (ζ).

The two parameters associated with the screed dynamics were obtained from the calibration provided in the base study: damping ratio of ζ = 1 and frequencies of ω_n_ = 0.045, ω_n_ = 0.065, and ω_n_ = 0.075 for simulations of one, two, or three passes of the paver, respectively.

(4) Differential thickness correction due to final roller compaction. This model reflects the proportional relationship between the ‘lift thickness’ laid by the paver (y_LIFT_) and the final ‘compacted thickness’ after rolling (y_COMP_).

The final elevation profile of the laid asphalt layer (y_LIFT_) will be given by the profile resulting from the total screed response (y = y_1_ + y_2_) and the lift thickness (h_LIFT_) established in the initial position. The resulting longitudinal profile must be corrected to incorporate the final compaction by the rollers. Equation (5) presents the expression for the final compacted elevation profile.

(5) $$y_{COMP}=\left(y+h_{LIFT}\right)\left(\frac{1}{1+FF}\right)+u\left(\frac{FF}{1+FF}\right)$$

where

h_LIFT_: Lift thickness or ‘loose’ thickness of asphalt mix laid by the paver.

FF: Fluff Factor representing proportional reduction in thickness due to compaction.

### 2.2. Control Principle of Automatic Levelling System

In an attempt to smooth out the vertical movements of the tow point during paving, paver manufacturers have introduced automatic screed control systems. The objective of these control systems is to keep the elevation of the tow point constant in relation to a reference despite the movement of the tractor unit as it travels over the surface being paved. Maintaining a steady elevation of the tow points enables the screed to keep a more uniform angle of attack, resulting in a smoother mat behind the screed [15].

**Figure 2:**
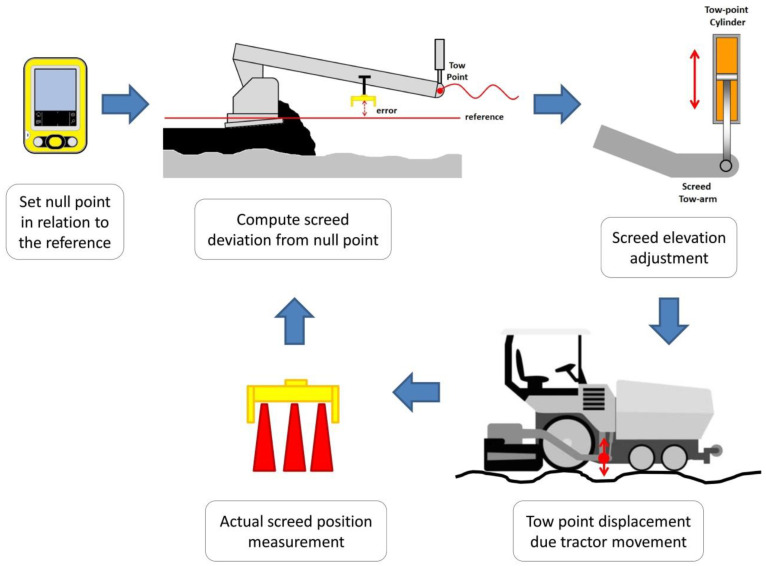
Levelling system working principle.

The system keeps the set elevation in relation to the reference (or null point) by means of an electrical sensor. If the sensor deviates from the null point during paving an electronic signal is transmitted to the tow-point solenoid valve, which allows the tow-point cylinder to raise or lower in order to restore the null point at the sensor (see Figure 2). Raising the tow point causes the screed’s angle of attack to rise, which increases math thickness. Lowering the tow point causes the screed’s angle of attack to decrease, which lowers the screed depth and layer thickness [16]. By operating in this way, deviations in the pavement surface are averaged out over the length of the reference.

### 2.3. Reference Types for Screed Control Systems

When using automatic screed control, the reference datum for the screed can be either fixed or mobile. The device used to sense or track the reference slope changes can be in-contact with the reference (mechanical sensors), contact-free acoustic sensor (ultrasonic), contact-free optical sensor (laser), or 3D navigation systems.

(1) Fixed references are used when building a certain profile prevails over other conditioning factors. It can be an erected string line, an adjacent pavement lane, an existing curb or any other tangible reference. A laser beam can also be used as a fixed reference when paving large areas where the sight distance is adequate and the pavement being placed has a constant longitudinal and transverse slope (such as parking lots, airport runways or port storage areas). Additionally, the latest 3D navigation systems for pavers allow project elevations to be preloaded into the controller and be used as a fixed reference for the paver screed. All the fixed references have in common the fact that they are independent of the surface being paved, so if the base condition is irregular with deep depressions and high points, the new mat behind the screed will contain areas of varying thickness of material as a result of these undulations in the base layer.
(2) When the main objective is paving for smoothness, or build rideability, the use of dynamic references is convenient, which are designed to regularize the underlying surface through long-distance averaging. This type of system is based on a longitudinal levelling device physically connected to the paver that moves alongside the tractor during paving. Various paver manufacturers have implemented different types of mobile reference devices to extend the relative wheelbase for the automatic screed control system. The operation of these reference systems, however, is essentially the same. The purpose of the mobile reference is to average the deviations in the existing surface over a distance that is greater than the wheelbase of the tractor unit itself. Earliest mobile references correspond to a rigid longitudinal instrument (levelling beam or tube) that rests directly on the surface being paved and slides over it in the form of a ski. Most recent mobile references use contactless acoustic sensors set-up over a floating beam attached to the paver.

The regularization capacity of the mobile reference will be determined by the length of the instrument used to carry out the averaging. The longer the reference used, the better the paver will average out profile variations in the existing pavement surface. Unlike fixed references, a mobile reference will not, however, ensure that the mix being placed is at a pre-fixed elevation. For the present study the use of a mobile contactless levelling-beam type reference was assumed.

### 2.4. Sensor Placement in Relation to the Screed

How quickly the screed responds to a change in the null point at the sensor is determined by the tracking sensor position in relation to the screed, which will affect either the profile or the smoothness built. The tow point serves as a point to raise or lower the tow arm in order to restore the null point at the sensor, and the sensor location becomes the screed control point. The distance from the control point (sensor) to the pivot point (screed trailing edge) becomes the effective tow arm length. Without the use of automatic levelling, the screed must travel five tow arm lengths to fully react to a correction; therefore, reducing the effective tow arm length through automatic control will shorten the distance (or reaction time) the paver needs to travel to complete a correction [16].

**Figure 3:**
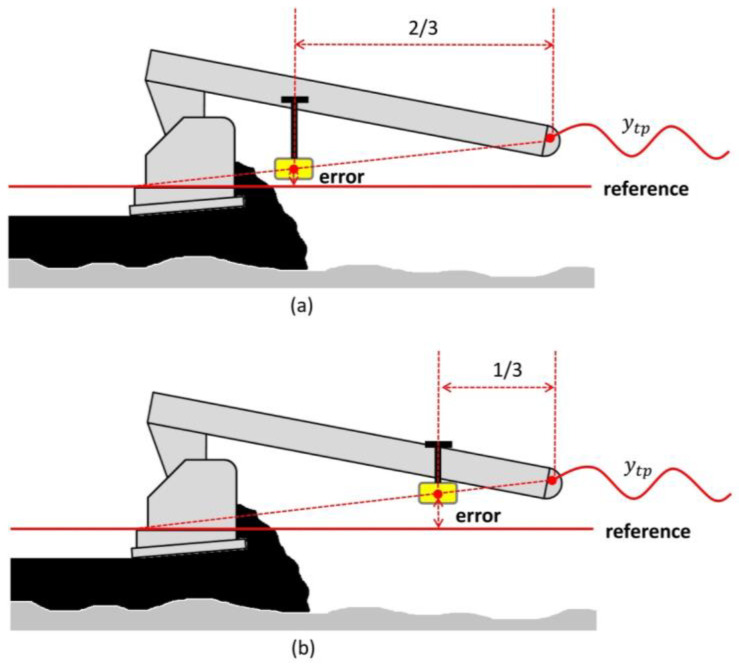
(**a**) Sensor placement toward the screed. (**b**) Sensor placement toward the tow point.

According to the above, moving the sensor towards the screed speeds up the reaction time. A more reactive screed would be beneficial when it is important to match a fixed reference (build profile), such as tracking a string line or matching a curb. For this application, the manufacturer recommends locating the sensor at a point 2/3 to 3/4 away from the tow point (relative to tow-arm length; see Figure 3a).

Moving the sensor towards the tow point slows down the reaction time. When smoothness is desired (build rideability) a less reactive screed is necessary, such as when utilizing a mechanical or non-contact levelling beam. For this application, manufacturers recommends to locate the sensor at a point 1/4 to 1/3 away from the tow point [15,16,17] (See Figure 3b).

## 3. Model Formulation

Taking into account the background information, an asphalt paver model is proposed to estimate the as-built IRI roughness when using an automatic screed control system through a levelling-beam mobile reference. The paver model was obtained by enhancing the base model to incorporate the following aspects of the automatic levelling system operation:

1. Reference profile obtained by the levelling beam as a result of the mobile long-base averaging.
2. Depending on the difference between the reference and the actual screed position at the sensor, the control unit will determine the proportional current input for the electro-valve.
3. The electro-valve time response to current input and hydraulic oil flow into the tow-point cylinder.
4. Lastly, depending on the oil flow direction, the cylinder will move up or down to control the tow-point position in relation to the reference value.

These aspects of the construction process constitute sub-models that will be integrated into a final model. Thereby, the developed paving model operates as a transfer function that, for an input given by the existing longitudinal profile, will produce an output corresponding to the new longitudinal profile of the rehabilitated pavement.

### 3.1. Levelling Beam Reference Profile

The model represents a contactless acoustic sensors system set-up over a floating beam attached to the paver. Typical floating beam reference systems for pavers are composed of three or four ultrasonic sensors mounted on a 9–13 m beam. Each sensor sends its measurement results to the central unit. In the control unit, the measured values are averaged, establishing a reference for the screed as a result of smoothing the underlying surface.

The tracking sensor (control point) should be located in the center of the length of the levelling beam (i.e., beam pivot point). By operating in this manner, the up and down movement of the beam during paving will not influence the averaged sensor values.

**Figure 4:**
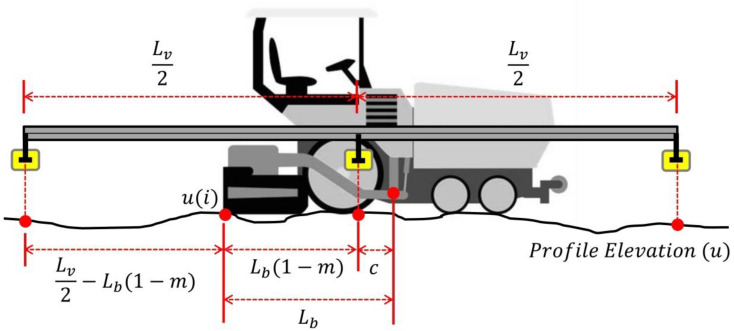
Reference profile calculation.

A 9 m long levelling beam with three sensors was assumed for modeling purposes. In this configuration, the center sensor is located in the middle of the levelling beam, and, therefore, it also coincides with the screed control point location (see Figure 4). The reference profile (u_lvl_) resulting from the long-base averaging beam is obtained by Equation (6).

(6) $$u_{lvl}=\frac{1}{3}\left\{u\left[i-\left(\frac{L_{v}}{2}-L_{b}(1-m)\right)\right]+u\left[i+L_{b}(1-m)\right]+u\left[i+\left(\frac{L_{v}}{2}+L_{b}(1-m)\right)\right]\right\}$$

where

L_v_: Levelling beam length. Assumed 9.0 m.

m: Location ratio of beam center in relation to the tow-arm length. M = c/L_b._

c: Distance from the tow point to the levelling beam center (center censor), in m.

i: Each of the ‘n’ elevation points of the longitudinal profile ‘u’ (i = 1, 2, …, n).

In Equation (6), the profile elevation ‘u’ is referenced with respect to the back of the paver (screed trailing edge).

### 3.2. Levelling System Control Unit

In the initial position, the offset of the control point in relation to the reference is set to zero, so no control deviation exists (null point). When the paver starts moving over the surface to be paved, the tow-point elevation and, therefore, screed position changes, thus deviating the sensor from the reference.

**Figure 5:**
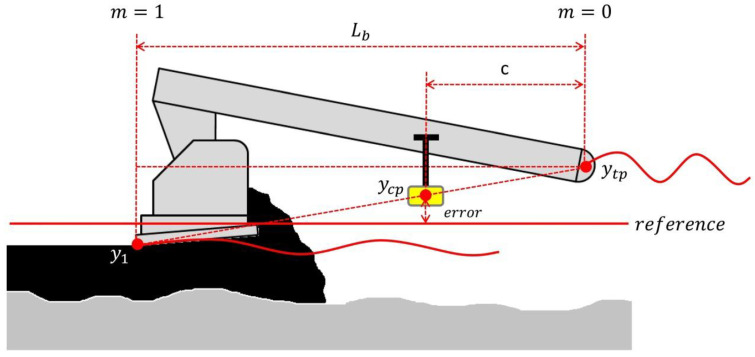
Screed control point location.

The control unit uses the elevation value established by the reference (target value), and compares it to the actual screed position at the control point; the deviation obtained is the error value (err) for the control signal. A direct relationship (Equations (7) and (8)) can be established between the tow-point elevation (y_tp_), screed position (y_1_) and control point elevation (y_cp_) (see Figure 5).

(7) $$\frac{y_{tp}-y_{1}}{L_{b}}=\frac{y_{tp}-y_{cp}}{c}\Rightarrow y_{cp}=y_{tp}\left(\frac{L_{b}-c}{L_{b}}\right)+\frac{c}{L_{b}}y_{1}$$

(8) $$m=\frac{c}{L_{b}}\Rightarrow y_{cp}=y_{tp}\left(1-m\right)+m\cdot y_{1}$$

Automatic levelling systems use sensitivity settings in order to establish how quickly, or aggressively, the controller reacts to a deviation. The settings range starts typically from 1 (low sensitivity) to 10 (high sensitivity). Behind this value, there is a pre-established combination of the control parameters “Dead band” and “Proportional band” [17,18,19].

Dead band (Db) is the resolution of the system, and defines the minimum deviation from the reference at the system will act upon. If a change in measurement is smaller than the dead band, the control unit will not commence any regulation. It serves the purpose of achieving a stable behavior of the screed around the control point. A given amount is necessary to allow for normal machine vibration.

The proportional band (Pb) is the range of the sensor reading above and below the target over which the hydraulic output signal varies proportionally to the deviation (error) from the target value. The minimum hydraulic output is applied when the sensor reading is just outside Db (beginning of the Pb) and the maximum hydraulic output is reached when the sensor reading deviates from the reference by an amount equal to or greater than the selected proportional band. A smaller Pb means a faster hydraulic response.

The proportional relationship between the amount of deviation at the sensor and the output signal of the controller is the Control Gain (Kc). Expressed in ratio of maximum valve current (i_v_/i_max_), the gain is given by Kc = 1/P_b_.

Control system manufacturers provide default sensitivity (SEN) values representing specific combinations of Db and Pb. Table 1 shows the values used in this study, corresponding to default settings provided for a proportional solenoid valve in a levelling system for asphalt pavers by the Mobile Automation Company [18].

**Figure 6:**
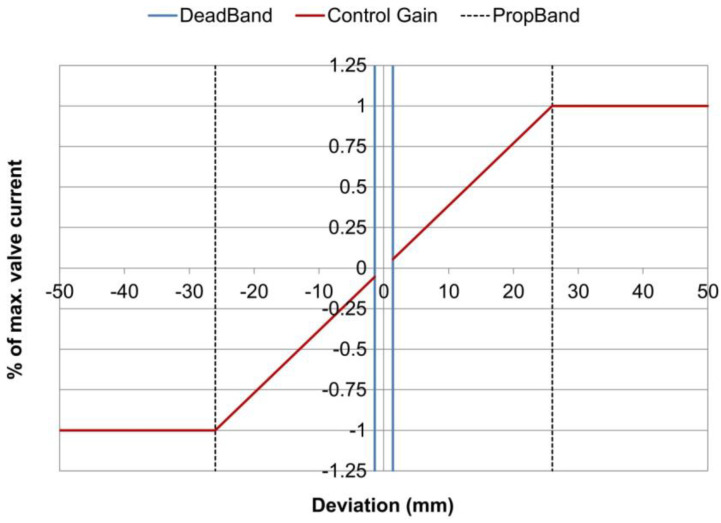
Control unit—dead band and prop band visualization.

As an example, when using a sensitivity value of SEN = 5, the control parameters are Db = 1.4 (mm), Pb = 26 (mm) and therefore gain Kc = 1/26 = 0.038 (1/mm) or Kc = 38 (1/m). This means that, for a deviation of err = 5 (mm) on the screed position in relation to the reference (evaluated at the control point), the control unit will send a command signal of i_c_ = 5 × 0.038 = 0.19 (or 19% of maximum valve current) to the solenoid valve. Any deviation greater than Pb = 26 mm will produce the maximum command signal of i_c_ = 1 (or 100% of maximum valve current). The relationship between the parameters can be seen graphically in Figure 6.

### 3.3. Control Valve Response

Asphalt pavers are provided with a control valve to adjust the tow-point position. The valve regulates the hydraulic oil flow into the tow-point cylinder chambers, which in turn produces the up-and-down motion of the piston that controls the screed tow-point elevation. The valves used for this purpose are typically electrically controlled solenoid valves. Depending on the solenoid actuator design, solenoid valves can provide for variable valve positioning and flow rate (proportional solenoid) or switch operation between fully open/closed valve positions (on–off solenoid). For this study, a typical proportional solenoid valve was assumed.

**Figure 7:**
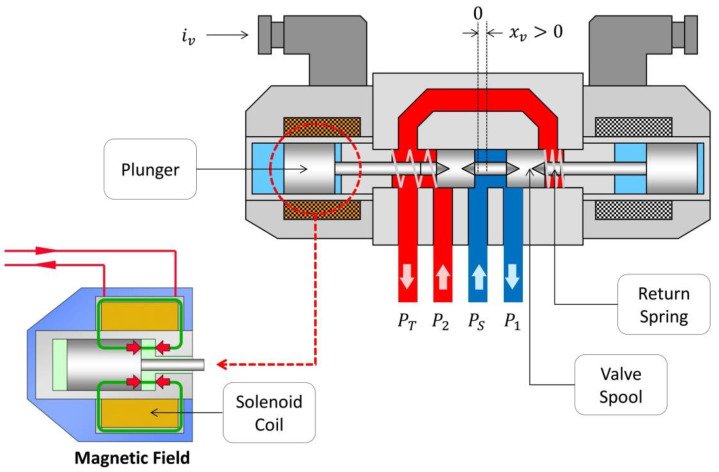
Solenoid valve working principle.

A proportional solenoid valve controls fluid flow rate by varying the size of the flow passage through the valve port orifice. Variable valve positioning is achieved by means of a solenoid actuator, which is an electric coil with a movable ferromagnetic core (plunger) in its center (see Figure 7).

The solenoid valve operation can be described by the interaction of three subsystems: (1) electro-magnetic subsystem, (2) mechanical subsystem, and (3) hydraulic subsystem.

In the closed or null position (de-energized state), a spring holds the spool against valve orifices, thus closing them and preventing flow. When the current flows through the solenoid, the coil is energized and creates a magnetic field (electro-magnetic subsystem). The magnetic field exerts an attraction force on the plunger (see Figure 7), moving it and overcoming the spring force (mechanical subsystem). The plunger moves the valve-spool thus opening the orifices and allowing flow through the valve (hydraulic subsystem). When de-energized, the return-spring pulls back the spool and plunger to the closed position.

The complete mathematical model of the valve solenoid actuator (electro-magnetic and mechanical subsystems) can be described by a third-order nonlinear system [20]. The dynamics of this system involve a large number of parameters, and many of them may only be known within some (wide) range, or even be completely unknown. Therefore, to develop simpler model approximations for practical applications, the valve manufacturer’s catalogue information usually provides well-known step responses and/or frequency responses for various sizes and types of valves [21].

For control system modelling purposes of this work, the third-order model for the solenoid actuator was simplified. When the valve dynamics are sufficiently fast, relative to the response of the process to be controlled (tow-point cylinder movement), the valve spool displacement (x_v_) from null can be considered proportional to a first-order filter on the input variable (current), which is accurate enough to model common proportional valves [22,23,24]. The simplified first-order model is presented in Equation (9).

(9) $${\dot{x}}_{v}=-\frac{1}{\tau_{v}}x_{v}+\frac{K_{i}}{\tau_{v}}\cdot i_{v}$$

where

x_v_: Valve spool displacement (m).

τ_v_: Equivalent valve time constant (s).

K_i_: Steady-state valve input-to-spool-position gain (m/A).

i_v_: Valve input current (A).

Applying the Laplace transform to Equation (9), assuming initial conditions equal to zero, and rearranging the terms, the transfer function of the system H_v_(s) is obtained by Equation (10).

(10) $$\frac{x_{v}\left(s\right)}{i_{v}(s)}=H_{v}\left(s\right)=\left(\frac{1}{\tau s+1}\right)K_{i}$$

The time constant (τ_v_) is obtained through the valve frequency. The proportional valve assumed for this work (Bosch-Rexroth 4/3-way control valve 4WRA-B6) has a step response switching time of 18 (ms) to ON and 20 (ms) to OFF. The valve frequency (f_v_) for one cycle (0-100%-0) is therefore obtained by Equation (11).

(11) $$f_{v}=\frac{1}{t_{on}+t_{off}}=26.3\;(Hz)$$

Then, the equivalent valve time constant is calculated in Equation (12) [22].

(12) $$\tau_{v}=\frac{1}{2\cdot \pi \cdot f_{v}}=6\;(ms)$$

The final aspect to be modelled is the relation between the spool position (x_v_) and the volume flow through valve orifices (hydraulic subsystem). For a matched and zero-lapped valve, the port opening area (A_v_) is proportional, to a good first approximation, to spool displacement ($A_{v}\approx w\cdot x_{v}$), which is also proportional to the current applied ($x_{v}\approx K_{i}\cdot i_{v}$). The tank (return) pressure is usually neglected in comparison to the line pressures ($P_{T}\approx 0$). Then, for positive spool displacement (x_v_ > 0), the two flow equations representing flow out of the valve (Q_1_) and flow back thought the valve (Q_2_) are given by the well-known orifice equation. As shown in Equation (13) and Equation (14) respectively [25].

(13) $$Q_{1}=C_{d}\cdot w\cdot x_{v}\sqrt{\frac{2}{\rho }\left(P_{s}-P_{1}\right)}=C_{d}\cdot w\cdot K_{i}\cdot i_{v}\sqrt{\frac{2}{\rho }\left(P_{s}-P_{1}\right)}=K_{f}\cdot i_{v}\sqrt{P_{s}-P_{1}}$$

(14) $$Q_{2}=C_{d}\cdot w\cdot x_{v}\sqrt{\frac{2}{\rho }\left(P_{2}-P_{T}\right)}=C_{d}\cdot w\cdot K_{i}\cdot i_{v}\sqrt{\frac{2}{\rho }P_{2}}=K_{f}\cdot i_{v}\sqrt{P_{2}}$$

The valve coefficient (K_f_) is given by Equation (15).

(15) $$K_{f}=C_{d}\cdot w\cdot K_{i}\sqrt{\frac{2}{\rho }}$$

where

Q_1_: Flow out of the valve through orifice port 1 (m^3^/s).

Q_2_: Flow back to the valve through orifice port 2 (m^3^/s).

w: Area gradient of the valve (m).

C_d_: Discharge coefficient.

P_s_: Supply pressure (Pa).

P_1_: Line pressure out of valve port 1 (Pa).

P_2_: Line pressure back to valve port 2 (Pa).

P_T_: Return tank pressure (Pa).

ρ: Hydraulic oil density (kg/m^3^).

For the control system analysis is necessary that these non-linear flow equations be linearized about an operating point (0) to obtain a linear transfer function. Equations (13) and (14) can be linearized by using a Taylor series expansion and neglecting the higher-order terms. Assuming a constant supply pressure (P_s_), the results of the linearized flow Equations (13) and (14) are presented in Equations (16) and (17), respectively.

(16) $${∆Q}_{1}=K_{q1}\cdot ∆i_{v}-K_{p1}\cdot ∆P_{1}$$

(17) $${∆Q}_{2}=K_{q2}\cdot ∆i_{v}+K_{p2}\cdot ∆P_{2}$$

where the flow-gain coefficients (K_q1_ and K_q2_) are obtained by Equations (18) and (19) and the flow-pressure coefficients (K_p1_ and K_p2_) are obtained by Equations (20) and (21), respectively.

(18) $$K_{q1}={\left.\frac{\partial Q_{1}}{\partial i_{v}}\right|}_{0}=K_{f}\sqrt{P_{s}-P_{10}}$$

(19) $$K_{q2}={\left.\frac{\partial Q_{2}}{\partial i_{v}}\right|}_{0}=K_{f}\sqrt{P_{20}}$$

(20) $$K_{p1}={\left.\frac{\partial Q_{1}}{\partial P_{1}}\right|}_{0}=\frac{K_{f}i_{v0}}{2\sqrt{P_{s}-P_{10}}}\;\;\;$$

(21) $$K_{p2}={\left.\frac{\partial Q_{2}}{\partial P_{2}}\right|}_{0}=\frac{K_{f}i_{v0}}{2\sqrt{P_{20}}}\;\;\;$$

The most important operating point is the valve null condition because system operation usually occurs near this region. The valve flow-gain is the largest and the flow-pressure coefficient is the smallest (K_p1_ = K_p2_ = K_p_ ≈ 0). Therefore, for a zero-lapped spool, the linearized flow-gain coefficient at the null condition (i_v0_ = 0 and P_10_ = P_20_ = P_s_/2) is given by Equation (22) [25].

(22) $$K_{q1}=K_{q2}=K_{q}=K_{f}\sqrt{\frac{P_{s}}{2}}$$

The valve coefficient (K_f_) may be determined experimentally, or it may be calculated using catalogue data from the rated condition test. For this test, the ports are connected via a restrictor to generate a load pressure differential (P_L_ = P_1_ − P_2_); therefore, Q_1_ = Q_2_. The load flow (Q_L_) equation under this condition can be expressed by Equation (23).

(23) $$Q_{L}=K_{f}\cdot i_{v}\sqrt{\frac{P_{s}-P_{L}}{2}}$$

Manufacturers quote the rated flow (Q_R_) at a rated current (i_max_). For proportional valves, the rated condition test is performed for a pressure drop ∆P_R_ = P_s_ − P_L_ = 1 MPa (10 bar). The valve coefficient is therefore calculated by Equation (24).

(24) $$Q_{R}=K_{f}\cdot i_{max}\sqrt{\frac{∆P_{R}}{2}}\Rightarrow K_{f}=\frac{Q_{R}}{i_{max}}\sqrt{\frac{2}{∆P_{R}}}$$

Combining the previous results with Equations (16) and (17), the linearized flow equations are obtained (valid near null point operation) by Equations (25) and (26).

(25) $$Q_{1}=\frac{Q_{R}}{i_{max}}\sqrt{\frac{2}{∆P_{R}}}\cdot \sqrt{\frac{P_{s}}{2}}\cdot i_{v}-K_{p}\cdot P_{1}=K_{Q}\cdot \frac{i_{v}}{i_{max}}-K_{p}\cdot P_{1}$$

(26) $$Q_{2}=\frac{Q_{R}}{i_{max}}\sqrt{\frac{2}{∆P_{R}}}\cdot \sqrt{\frac{P_{s}}{2}}\cdot i_{v}+K_{p}\cdot P_{2}=K_{Q}\cdot \frac{i_{v}}{i_{max}}+K_{p}\cdot P_{2}$$

where the valve flow-gain is expressed as: $K_{Q}=Q_{R}\cdot \sqrt{\frac{P_{s}}{∆P_{R}}}$.

For the asphalt paver, a proportional solenoid valve with rated flow of Q_R_ = 12 (L/min), a supply pressure of P_s_ = 5 (MPa) and a flow-pressure coefficient of K_p_ = 5 × 10^−11^ (m^5^/N·s) were assumed. Applying the Laplace transform to Equations (25) and (26), assuming initial conditions equal to zero, and rearranging the terms, the linear transfer functions are obtained in Equations (27) and (28).

(27) $$Q_{1}(s)=K_{Q}\cdot i_{c}(s)-K_{p}\cdot P_{1}(s)$$

(28) $$Q_{2}(s)=K_{Q}\cdot i_{c}\left(s\right)+K_{p}\cdot P_{2}(s)$$

where the input current is: $i_{c}=\frac{i_{v}}{i_{max}}$.

From Equations (27) and (28). it can be seen that input current is the ratio of maximum valve current (i_v_/i_max_), which is the command signal out of the control unit derived in the previous section.

### 3.4. Cylinder Dynamic Response

**Figure 8:**
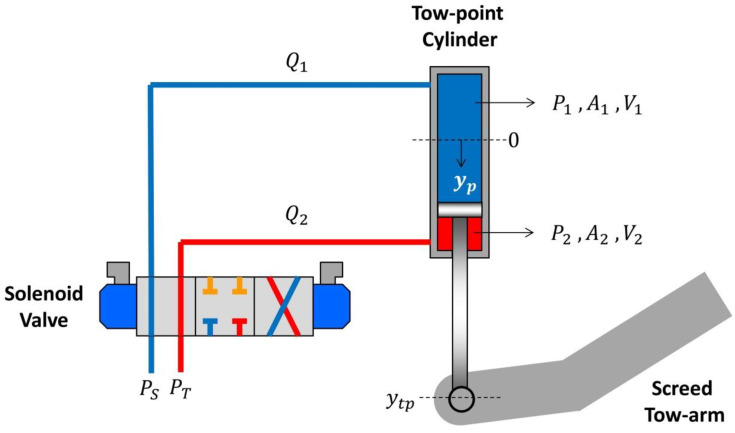
Solenoid valve–cylinder control system.

The tow-point hydraulic cylinder consists of a single rod and single ended piston (asymmetric cylinder) as shown in Figure 8. The model for the valve–cylinder system presented herein is based on the derivation procedure found in [24].

From Figure 8, P_S_ is the supply pressure from the hydraulic pump and P_T_ is the return pressure to the tank. Assuming there are no external forces acting on the tow point other than the lift or downforce exerted by the piston motion, the mathematical model of this system is derived in Equation (29) using force balance.

(29) $$m_{e}{\ddot{y}}_{p}\left(t\right)+b{\dot{y}}_{p}\left(t\right)=P_{1}\cdot A_{1}-P_{2}\cdot A_{2}$$

where

m_e_: Equivalent mass supported by the cylinder. Tow-arm + half screed = 800 (kg).

y_p_: Piston displacement inside the cylinder relative to initial position (m).

b: Piston viscous/coulomb friction damping coefficient (N·s/m).

P_1_: Fluid pressures in the upper cylinder chamber (Pa).

A_1_: Cylinder upper chamber area. Piston side (m^2^).

P_2_: Fluid pressures in the lower cylinder chamber (Pa).

A_2_: Cylinder lower chamber area. Rod side (m^2^).

The cylinder chambers areas A_1_ and A_2_ are calculated from the rod and piston diameters.

$$A_{1}=\pi {\left(\frac{D}{2}\right)}^{2}$$

$$A_{2}=\pi \left(\frac{D^{2}-d^{2}}{4}\right)$$

where

D: Piston diameter. Assumed 0.1 (m).

d: Rod diameter. Assumed 0.05 (m).

Applying the Laplace transform to Equation (29), assuming initial conditions equal to zero, and rearranging the terms, Equation (30) is obtained:

(30) $$y_{p}\left(s\right)\left(m_{e}s^{2}+bs\right)=A_{1}P_{1}\left(s\right)-A_{2}P_{2}(s)$$

The next step is to establish the flow-continuity Equations (31) and (32) for each cylinder chamber. Neglecting fluid leakage across the piston.

(31) $$Q_{1}=A_{1}\frac{{dy}_{p}}{dt}+\frac{V_{1}}{\beta_{e}}\cdot \frac{{dP}_{1}}{dt}$$

(32) $$Q_{2}=A_{2}\frac{{dy}_{p}}{dt}+\frac{V_{2}}{\beta_{e}}\cdot \frac{{dP}_{2}}{dt}$$

where

β_e_: Effective bulk modulus of the hydraulic oil. Assumed 700 (MPa).

The initial or mid-stroke position of the piston (y_p_ = 0) is defined in such a way that the volumes of both cylinder chambers are equal (V_10_ = V_20_). Assuming small piston displacements within the vicinity of the mid-stroke, the approximation shown in Equation (33) is used [24].

(33) $$\frac{V_{1}}{\beta_{e}}\approx \frac{V_{2}}{\beta_{e}}\approx \frac{1}{\beta_{e}}\left(\frac{V_{10}+V_{20}}{2}\right)=\frac{1}{\beta_{e}}\left(\frac{V_{t}}{2}\right)=C_{H}$$

where

V_t_: Total cylinder volume at mid-stroke position (m^3^).

C_H_: Hydraulic capacitance (m^3^/Pa).

Thus, Equations (31) and (32) can be re-written in the Laplace domain as Equations (34) and (35), respectively.

(34) $$Q_{1}\left(s\right)=sA_{1}\cdot y\left(s\right)+sC_{H}\cdot P_{1}(s)$$

(35) $$Q_{2}\left(s\right)=sA_{2}\cdot y\left(s\right)+sC_{H}\cdot P_{2}(s)$$

Combining Equations (34) and (35) with the linearized valve-flow Equations (27) and (28) and rearranging the terms, Equations (36) and (37) are obtained in the Laplace domain for pressures in the upper and lower cylinder chambers.

(36) $$P_{1}\left(s\right)=\frac{K_{Q}}{sC_{H}+K_{p}}\cdot i_{c}(s)-\frac{sA_{1}}{sC_{H}+K_{p}}$$

(37) $$P_{2}\left(s\right)=-\frac{K_{Q}}{sC_{H}+K_{p}}\cdot i_{c}\left(s\right)+\frac{sA_{2}}{sC_{H}+K_{p}}$$

Replacing these results in the force-balance Equation (30) and rearranging the terms, Equation (38) for the valve–cylinder system is obtained.

(38) $$y_{p}\left(s\right)\left[\left(m_{e}s^{2}+bs\right)\left(sC_{H}+K_{p}\right)+s\left({A_{1}}^{2}+{A_{2}}^{2}\right)\right]=K_{Q}\left(A_{1}+A_{2}\right)\cdot i_{c}(s)$$

The transfer function H_C_(s) of the system is deduced from Equation (38), neglecting the friction damping (b) and defining the rod/piston area ratio: α = A_2_/A_1_.

(39) $$\frac{y_{p}(s)}{i_{c}(s)}=H_{c}(s)=\frac{1}{s}\cdot \frac{\frac{1+\alpha }{A_{1}\left(1+\alpha^{2}\right)}\cdot K_{Q}}{\frac{m_{e}C_{H}}{{A_{1}}^{2}\left(1+\alpha^{2}\right)}s^{2}+\frac{m_{e}K_{p}}{{A_{1}}^{2}\left(1+\alpha^{2}\right)}s+1}$$

Applying the substitutions in Equations (40) and (41), Equation (39) is then rewritten in terms of system natural frequency (ω) and damping ratio (ζ), and the final transfer function of the system H_C_(s) is obtained as Equation (42).

(40) $$\omega =\sqrt{\frac{{A_{1}}^{2}\left(1+\alpha^{2}\right)}{m_{e}C_{H}}}$$

(41) $$\zeta =\frac{m_{e}K_{p}}{2\sqrt{m_{e}C_{H}}\cdot \sqrt{{A_{1}}^{2}\left(1+\alpha^{2}\right)}}$$

(42) $$H_{c}(s)=K_{Q}\cdot \frac{\frac{1+\alpha }{A_{1}\left(1+\alpha^{2}\right)}}{\frac{1}{\omega^{2}}s^{2}+\frac{2\zeta }{\omega }s+1}\cdot \frac{1}{s}$$

The transfer function obtained in Equation (42) for the valve–cylinder system, although being relatively simple, has been used in other levelling-system-related research [26,27,28]; therefore, it was considered accurate enough for this work.

### 3.5. Models Integration

**Figure 9:**
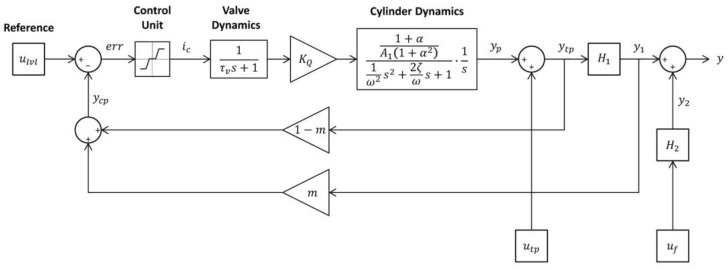
Blocks diagram—final model for screed response.

The integration consists of coupling the base model with the levelling system model and their respective input variables. The integration was made in the Matlab/Simulink environment. Figure 9 shows the blocks diagram representation of the final model for the screed response (y). The resulting longitudinal profile is then corrected to incorporate proportional layer thickness reduction due to final roller compaction (see Equation (5)).

In Figure 9, the reference profile (u_lvl_) resulting from the levelling beam sub-model is the input to the control system. The difference, or deviation, of the control point position (y_cp_) in relation to the reference is the error value (err) for the control signal. The error value is used by the control unit sub-model to provide a proportional current input (i_c_) for the electro-valve. The valve response sub-model will produce a spool-displacement and flow-gain (K_Q_) proportional to current input. Depending on the oil-flow rate from the valve into the cylinder chambers, the cylinder response sub-model will move the piston (y_p_) up or down to control the tow-point position and therefore screed elevation. The tow-point position (y_tp_) is the result of piston displacement and tow-point trajectory (u_tp_) due to paver motion given by the base model. The screed position (y_1_) in response to tow-point movement is given by the screed reaction time base model (H_1_). The tow-point position and screed position is then used to calculate the new control point elevation (y_cp_) and re-evaluate the error value for the control system, which will operate as a continuous loop during the paving simulation. On each step, the total screed response (y) is given by the addition of screed trajectory (y_1_) and screed vibration (y_2_) sub-models H_1_ and H_2_, respectively (base model).

## 4. Model Calibration

Parameters related to certain paver characteristic dimensions (L_b_, L_axl_, L_wb_) and the paving process (V, h_LIFT_, FF) will determine the proposed model response. These parameters are either known or can be approximated using typical values used on paving projects. The two parameters associated with the screed dynamic model were obtained from the calibration provided in the base study [11]: a damping ratio of ζ = 1 and natural frequencies of ω_n_ = 0.045, ω_n_ = 0.065, and ω_n_ = 0.075 for simulations of one, two, or three passes of the paver, respectively.

**Figure 10:**
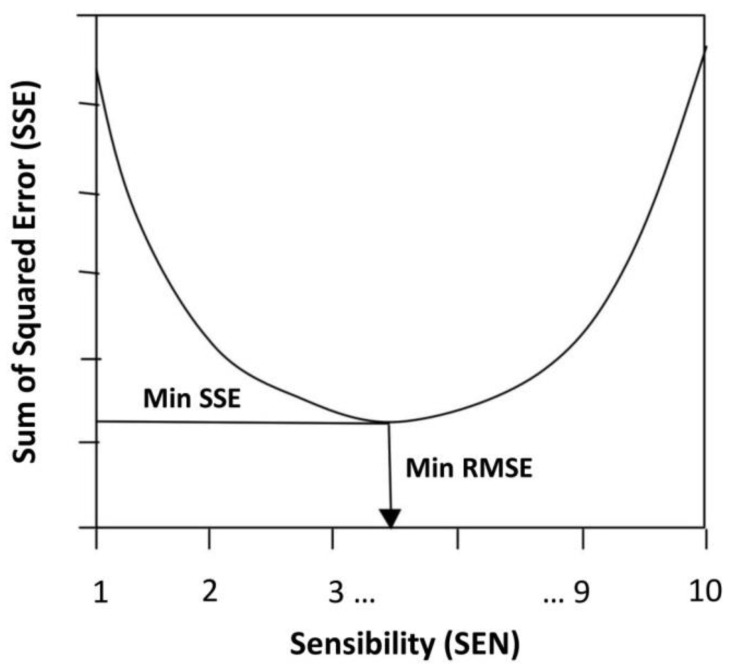
Calibration method—minimization of SSE.

Therefore, the unknown parameters to be determined are the levelling system sensibility (SEN) and the ratio of sensor location ‘m’ in relation to the tow-arm length. The calibration procedure is based on identifying the model sensibility and control point location values that produce the best fit between the IRI computed over the simulated as-built profile and the real as-built IRI data observed for the same initial longitudinal profile. The process of optimization involved the minimization of the Sum of Squared Errors (SSE) resulting from the difference between the observed and estimated IRI values. By minimizing the SSE value, the Root-Mean-Square Error (RMSE) of the estimation is reduced, which implies finding the calibration factor that guarantees the best fit for the predicted IRI and, consequently, the model’s calibration (See Figure 10).

### 4.1. Database for Calibration

To calibrate the model, comprehensive data on overlay works are needed. This includes longitudinal profile data measured both before and after works, as well as background information on the paving process related to the model parameters (paver characteristics, laydown thickness, etc.). Three requirements have to be met by the pavement works data chosen for calibration:

(1) The existing surface was not milled or excessively repaired before overlay construction; as a result, the as-built profile data are highly related to the paver leveling response to the original surface profile. (2) A wheeled paver was used for laying the asphalt overlay. (3) To ensure that the model calibration accurately reflects the screed automatically controlled response, the paving work must be executed utilizing an automatic screed control system through a mobile levelling beam.

The information available from the SPS-5 experiment of the North American Long-Term Pavement Performance (LTPP) program served as the data source for the analysis. The SPS-5 experiment was specially designed to assess the impact of various overlay alternatives on the pavement performance. A first pre-selection process of site candidates to be used in the calibration was conducted after a thorough review of the 210 sections available in the database. All sections that involved milling prior to overlay construction (intensive previous repair) and/or used a tracked paver for the laydown were excluded.

Additionally, some sections were discarded because they had insufficient information or due to differences between the design and as-built project; for instance, sections designed as minor surface repair prior to overlay in which milling was actually performed, or the lack of a profile evaluation before or after the paving works. The pre-selection resulted in 32 candidate sections.

Only the works that employed automated levelling during paving were chosen from the pre-selection. For some sections, this information was directly provided in the construction report; in other cases, it was deducted from the longitudinal profile power spectral density (PSD) analyzed before and after the overlay. Additional information and findings about the PSD analysis are available in the base study [11].

Lastly, some sections showed an after-overlay roughness value (IRI-post) greater than the existing roughness prior to overlay construction (IRI-pre). This inconsistent data affects the calibration process accuracy, and occurs likely due to inadequate construction quality, or to a lesser extent, potential survey errors. Given that the proposed model relies on a number of idealized construction process assumptions, such as constant speed and homogeneity of the mixture, which can be replicated (approximately) on the job site with good paving practices, it seem reasonable to remove from the analysis the sections in which the IRI-post (calculated at 100 m intervals) increased or showed a decrease of less than 0.1 m/km in comparison to the IRI-pre. Therefore, the analysis only took into account those sections that showed an average IRI drop of more than 0.1 m/km after overlay construction.

The selection process led to 15 sections suitable to be used in calibration. Table 2 presents a summary of the finally selected data. The “compacted thickness” (h_COMP_) and “lift thickness” (h_LIFT_) informed for each paving work were used to compute the Fluff Factor (FF).

Sections having SHRP ID codes 502, 503, 504, and 505 indicate minor surface preparation—that is, localized patching without milling—prior to overlay construction. Code sections 504 and 505 are overlays with virgin mix in thick and thin thickness (5.1 cm and 12.7 cm, respectively); code sections 502 and 503 correspond to overlay works with recycled HMA mix in the same thin and thick thicknesses, respectively. In sections 503 and 504 (thick thickness), the overlay was applied in two or three consecutive paver passes, which will be replicated on the simulations in order to calibrate the model.

A review of manufacturer catalogs was used to establish the dimensions of the paver models used for calibration. This information was gathered using three complementary approaches: (1) the value was directly available for the required paver model in the catalog, (2) estimation based on other dimensions listed in the catalog, and (3) dimension calculated using average values derived from other catalogs for a similar paver type. The results of the asphalt pavers dimensions used in the calibration are listed in Table 3.

### 4.2. Calibration Process

The calibration procedure involved computing the longitudinal profiles derived from the laydown simulation for every paving work, using the pre-overlay profile and the previously defined model values as input parameters. The longitudinal profiles surveyed in the internal (left, L) and external (right, R) wheel paths of the road lane, recorded before and after paving, were analyzed for each of the 15 overlay sections, totaling 60 longitudinal profiles for calibration.

Since the database lacked information on paving speed, a standard speed of V = 5 m/min—regarded as a typical value in the paving practice—was assumed for all simulations. In relation to the parameters to be calibrated; four control point positions (m = 1/4, m = 1/3, m = 2/3, and m = 3/4) and SEN values ranging from 1 to 10 (see Table 1) were assumed. This resulted in a total of 40 simulations for each paver pass.

The IRI computed over 20 m intervals was selected as the analysis unit, in order to maximize the sample size. The standard length of each LTPP segment is 152 m resulting in seven IRI values per profile and 210 post-overlay real IRI data for calibration. For paving projects where the layer was laid over multiple paver passes, the longitudinal profile derived from the first pass simulation served as the input for the second pass simulation. The same process was followed for sections where the laydown was completed in three layers, leading up to 2320 simulations that were programmed in Matlab^®^ software.

The IRI for the left and right wheel paths, as well as the average value between wheel paths, were computed every 20 m using the profiles obtained in each simulation. The prediction square error (SE) of each simulation was calculated by comparing all of the 40 average IRI values (10 SEN values per four control positions) obtained in each section against the corresponding IRI computed from the real overlay as-built profiles. The combination of sensibility (SEN) and sensor position ‘m’ that results in the lowest SSE calculated, considering all sections, is the one that minimizes the paver model estimation error RMSE.

## 5. Results

### 5.1. Optimal Sensibility and Set-Point Placement

**Figure 11:**
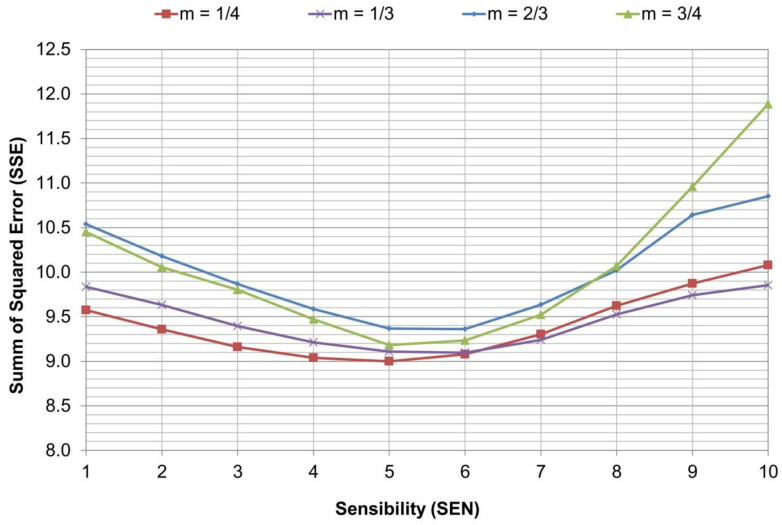
Calibration results—minimization of SSE.

Figure 11 shows the results of the SSE for each simulated Sensibility (SEN) and sensor placement values. It can be seen that the optimal SEN value is in the range of 4–6 for any control point configuration (m = 1/4, m = 1/3, m = 2/3 and m = 3/4). These results are consistent with control system manufacturer’s recommendations about middle sensibility settings; thus, the screed reacts to the change in grade quickly enough to achieve the correction, but without “overshooting” the new elevation [18,19].

The worst SSE results of the calibration are for higher SEN values, which is consistent with the levelling system working principle. In high SEN settings, the dead band is very small (Db = 0.4 mm for SEN = 10) so the control unit is constantly detecting an error at the sensor and sending a command signal to the tow-point cylinder; furthermore, the proportional band (Pb) is the smallest (Pb = 1 mm for SEN = 10), which means a faster hydraulic response. Thus, the levelling system is over-correcting the screed position all the time which in turn affects smoothness negatively.

Regarding sensor position, the best calibration results were obtained for m = 1/4 and m = 1/3, which is in accordance with the recommendations when using a mobile levelling beam [15,16,17].

According to Figure 11, the optimal value corresponds to an automatic levelling system sensibility of SEN = 5 with a center sensor position of m = 1/4 away from the tow point, associated with an SSE = 9.0 that minimizes the RMSE to a value of 0.30 m/km for the predicted IRI. For the sensor position of m = 1/3, the best sensibility is SEN = 6 with almost the same results (SSE = 9.1 and RMSE = 0.30 m/km). This is actually the factory default setting of the MOBA system used as reference for the sensibility table.

**Figure 12:**
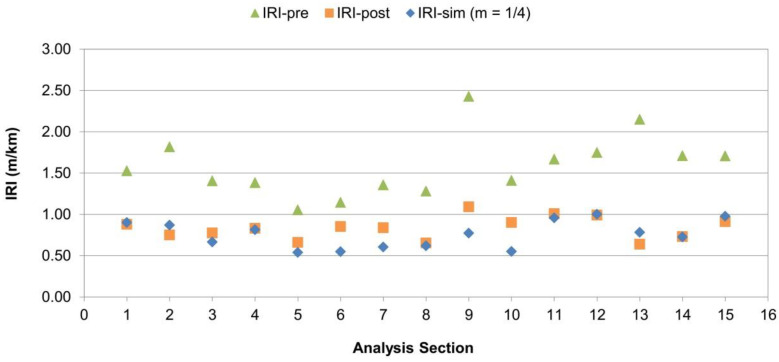
Simulation results—IRI @ 100 m comparison.

It is standard procedure in pavement quality control and supervision to report roughness surveys at longer intervals because IRI data every 20 m could be highly scattered around the representative (mean) roughness value for the segment. Therefore, a 100 m evaluation interval was employed for this purpose, which is regarded as a common standard in several countries [29]. From the results obtained, the IRI was calculated every 100 m for each section considering the optimal SEN and sensor placement determined in the calibration, that is, using values of SEN = 5 and m = 1/4. The results obtained are presented in Figure 12.

From Figure 12, it can be observed that the predicted values (IRI-sim) are always lower than the values prior to the overlay construction (IRI-pre), which demonstrates the levelling effect of the laydown process. Additionally, in general, the predicted value is consistent with the real IRI value observed after works (IRI-post), with a mean estimation error RMSE of 0.17 m/km equivalent to a Mean Absolute Percentage Error (MAPE) of 15.1%.

### 5.2. Comparison against Base Model

**Figure 13:**
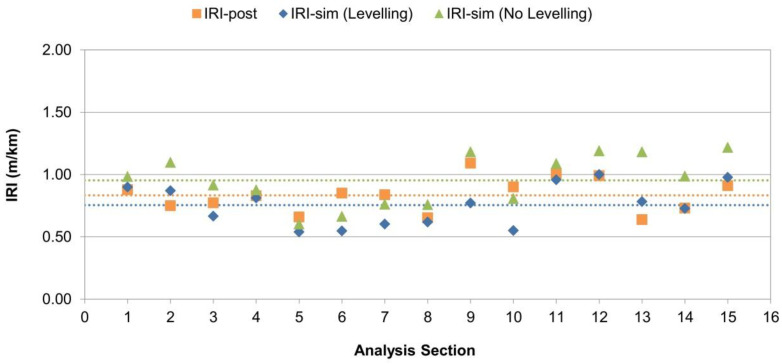
Models comparison—with and without levelling system.

For assessing the improvement of the proposed model against the base model, the model of wheeled paver without automatic levelling was tested with the same data set used herein. The results for the 15 study sections are presented in Figure 13.

Figure 13 shows that when using the base model, the predicted IRI-sim values (No Levelling) are in average higher than the real IRI-post values observed after works, which indicates that the base model overestimates as-built IRI on works that uses automatic levelling system (as expected). The mean estimation error is RMSE = 0.22 m/km equivalent to a MAPE of 22.6%.

When using the model with automatic levelling the predicted IRI-sim values (Levelling) are more consistent with real as-built IRI values, showing closer average results between them and lower error (RMSE = 0.17 m/km and MAPE = 15.1%). Additionally, the IRI-sim values of the enhanced model with automatic levelling are always lower than the IRI-sim values obtained from the base model (No Levelling), which demonstrates the additional levelling effect provided by the automatic control system. According to these results, the enhanced model represents an improvement over the base model, demonstrating the potential of the proposed model as a prediction tool for as-built roughness IRI when using a levelling-beam type reference for assisted levelling.

### 5.3. Proposed Model Profiles Visualization

**Figure 14:**
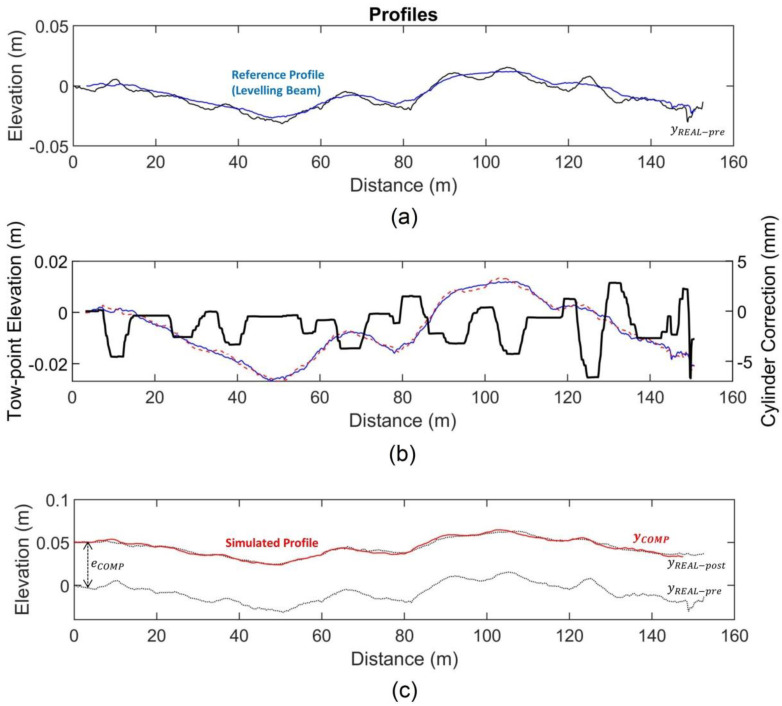
Model results graphical visualization. (**a**) Levelling beam reference. (**b**) Sensor error and cylinder correction. (**c**) Final simulated profile.

The model developed allows a graphical representation of the simulated aspects of the construction process. In this regard, the results of the different sub-model can be plotted independently during simulation to visualize their effect on the profile transformation. As an example, the paving modelling for the right wheel path of the New Jersey 0502 section is presented. According to Table 2, this section corresponds to an overlay constructed in a single layer with lift thickness h_LIFT_ = 6.1 cm (2.4 in) using a Barber-Greene BG-260 paver. The compacted thickness was h_COMP_ = 5.1 cm (2.0 in), equivalent to a Fluff Factor FF = 0.20. According to the base study, a natural frequency ω_n_ = 0.045 for the single layer was used. For the levelling system, sensibility SEN = 5 and sensor placement m = ¼ obtained from the calibration were used. The simulation results are presented in Figure 14.

Figure 14a shows the levelling beam reference profile (blue line) resulting from the long-base averaging of the existing surface profile. It can be seen that the longest wavelength associated with road vertical alignment is still present, but wavelengths shorter than the levelling-beam length (9 m) are smoothed out. Figure 14b shows in the left axis the sensor position (dashed red) in relation to the reference (blue). Axis limits are adjusted so elevation changes can be seen. The right axis (black) shows the cylinder correction movement (mm) acting on the tow point.

As can be seen, when the sensor position is above the reference, the cylinder moves down to lower the tow point and therefore screed position. When the error is less than Db (2.4 mm), the cylinder does not act and remains stable; otherwise, when the sensor is below the reference, the cylinder moves up to raise the tow point and screed position. Figure 14c shows in red the final simulated profile (y_COMP_) after the proportional thickness correction due to roller compaction (coefficient FF = 0.20).

The roughness of the existing profile is IRI-pre = 1.66 m/km (calculated over 100 m intervals), and real overlay as-built roughness is IRI-post = 0.97 m/km. The roughness of the final profile obtained from the simulation is IRI-sim = 0.95 m/km, corresponding to a prediction error of ∆IRI = 0.02 m/km.

## 6. Discussion

In a previous study by the authors, an as-built IRI prediction model for asphalt overlays based on profile transformation was proposed. The model, used as basis for this study, was developed for the case of wheeled pavers without automatic screed levelling.

In the base model, as well as in the real paving process, the paver screed decreases its levelling efficiency as the wavelength of the defects causing the roughness increases, which is the reason why pavers use automatic levelling systems to regulate the tow-point elevation and compensate for the lowest levelling efficiency at longer wavelength defects. In this work, further development of the base model is presented, including the effects of using an automatic screed control system through a levelling beam type mobile reference.

The proposed model describes an idealized construction process as a result of the integration of four sub-models:

1. Modelling of the reference profile obtained by the levelling beam as a result of the mobile long-base averaging.
2. Command signal model. The error value calculated as the difference between the reference and the actual screed position at the sensor is used by the control unit to provide a proportional current input for the electro-valve.
3. Electro-valve response model. The valve was represented by a first-order linear system time response and flow-gain proportional to current input. The result of this model is the hydraulic oil flow into the tow-point cylinder chambers.
4. Hydraulic cylinder model. Depending on the oil-flow rate from the valve into the cylinder chambers, the piston will move up or down to control the tow-point position and therefore screed elevation.

The model was calibrated from a selection of SPS-5 experiment sections from the LTPP database. The results of IRI every 100 m computed from the simulations show a mean estimation error RMSE of 0.17 m/km for the predicted IRI, equivalent to a mean percentage error MAPE of 15.1% with respect to the real IRI values observed after overlay construction.

The model developed involve a novel methodological approach for the prediction of as-built IRI, and the results obtained represent an improvement with respect to the base model, demonstrating the potential of the proposed approach as a valuable modeling alternative.

## Figures and Tables

**Table 1:** Sensitivity table for proportional and servo valves.

Sensitivity (SEN)	Dead Band Db (mm)	Prop Band Pb (mm)
1	2.2	46.0
2	2.0	41.0
3	1.8	36.0
4	1.6	31.0
5	1.4	26.0
6	1.2	21.0
7	1.0	16.0
8	0.8	11.0
9	0.6	6.0
10	0.4	1.0

Source: MOBA-Matic Levelling System for Paver.

**Table 2:** LTPP SPS-5 sections for model calibration.

SPS-5 Test Site			ASPHALT PAVER		LIFT_NO	$h_{LIFT}$ cm (in)	$h_{COMP}$ cm (in)	Fluff Factor FF
STATE_CODE	STATE_CODE_EXP	SHRP_ID	Manufacturer (**)	Model				
8	Colorado	0502	BARBER GREENE	BG-260B	1	7.9 (3.1)	6.4 (2.5)	0.24
8	Colorado	0503	BARBER GREENE	BG-260B	1	8.9 (3.5)	6.9 (2.7)	0.30
					2	6.4 (2.5)	4.8 (1.9)	0.30
8	Colorado	0504	BARBER GREENE	BG-260B	1	10.4 (4.1)	6.4 (2.5)	0.63
					2	10.7 (4.2)	6.6 (2.6)	0.63
8	Colorado	0505	BARBER GREENE	BG-260B	1	7.4 (2.9)	6.4 (2.5)	0.16
23	Maine	0502	BLAW-KNOX	PF-180H	1	6.1 (2.4)	5.3 (2.1)	0.14
					2	4.1 (1.6)	3.6 (1.4)	0.14
23	Maine	0503	BLAW-KNOX	PF-180H	1	5.3 (2.1)	4.6 (1.8)	0.17
					2	5.1 (2.0)	4.6 (1.8)	0.11
					3	5.8 (2.3)	5.1 (2.0)	0.15
23	Maine	0504	BLAW-KNOX	PF-180H	1	5.8 (2.3)	5.1 (2.0)	0.15
					2	4.8 (1.9)	4.3 (1.7)	0.12
					3	6.1 (2.4) (*)	4.8 (1.9)	0.25
23	Maine	0505	BLAW-KNOX	PF-180H	1	3.8 (1.5)	3.0 (1.2)	0.25
					2	3.8 (1.5)	3.0 (1.2)	0.25
24	Maryland	0503	BARBER GREENE	BG-240	1	5.1 (2.0)	4.1 (1.6)	0.25
					2	5.1 (2.0)	4.1 (1.6)	0.25
					3	6.1 (2.4)	5.1 (2.0)	0.20
24	Maryland	0504	BARBER GREENE	BG-240	1	5.1 (2.0)	3.8 (1.5)	0.33
					2	5.1 (2.0)	3.8 (1.5)	0.33
					3	6.6 (2.6)	5.1 (2.0)	0.30
24	Maryland	0505	BARBER GREENE	BG-240	1	6.4 (2.5)	5.3 (2.1)	0.19
34	New Jersey	0502	BARBER GREENE	BG-260	1	6.1 (2.4)	5.1 (2.0)	0.20
34	New Jersey	0503	BARBER GREENE	BG-260	1	8.9 (3.5)	7.6 (3.0)	0.17
					2	6.4 (2.5)	5.1 (2.0)	0.25
34	New Jersey	0504	BARBER GREENE	BG-260	1	10.2 (4.0)	7.6 (3.0)	0.33
					2	6.4 (2.5)	5.1 (2.0)	0.25
34	New Jersey	0505	BARBER GREENE	BG-260	1	6.4 (2.5)	5.1 (2.0)	0.25

(*) No ‘lift thickness’ information reported. Assumed from the general rule of about 1/4″ roll-down per 1″ of screed laid thickness (FF = 0.25). (**) Barber-Greene, A division of Caterpillar Paving Products Inc., Montgomery, IL, USA.; Blaw-Knox Construction Equipment Co., Mattoon, IL, USA.

**Table 3:** Paver characteristics dimensions.

ASPHALT PAVER		$L_{axl}$ (mm)	$L_{wb}$ (mm)	$L_{b}$ (mm)	$L_{t}$ (mm)
MANUFACTURER	MODEL				
BARBER GREENE	BG-260B	850	2510	3415	610
BLAW-KNOX	PF-180H	914	2540	3330	610
BARBER GREENE	BG-240	850	2180	3150	610
BARBER GREENE	BG-260	850	2510	3415	610

Source: Own elaboration based on manufacturer’s catalogs.

## Data Availability

The database used in the study was derived from LTPP InfoPave webpage which is publicly available at https://infopave.fhwa.dot.gov/ (accessed on 19 March 2024).
